# Alleviation and prevention of severe allergic rhinitis and conjunctivitis following long-term lemon juice use: a case report

**DOI:** 10.4076/1757-1626-2-8971

**Published:** 2009-08-24

**Authors:** Konstantinos GI Vazouras, Jota Partheniou, Ioannis DK Dimoliatis

**Affiliations:** Department of Hygiene & Epidemiology, University of Ioannina School of Medicine, University Campus45110, IoanninaGreece

## Abstract

Allergic rhinitis (often coexisting with allergic conjunctivitis) is a highly prevalent, chronic, inflammatory disease. Most cases are not extraordinary; however, they may result in significant impairment in quality of life of patients, as well as in economical damage for both health-care system and patients. This case report describes the experiences of a middle-aged woman with the illness, who managed to completely alleviate and prevent her symptoms, in terms of intensity and chronicity, by drinking natural lemon juice diluted with water. Lemon changed her life radically.

## Introduction

Allergic rhinitis (AR) is a highly prevalent, allergen-induced, upper-airway inflammatory disease, characterized by hyperreactive airway mucosa and episodes of symptom chronicity with periods of acute exacerbation [1]. If AR is combined with conjunctivitis, the condition is termed allergic rhinoconjunctivitis (ARC). AR may be perennial, which means that patients have symptoms year round to allergens that have no seasonal variation.

The prevalence of AR is a controversial issue, as published prevalence rates differ significantly [2]. However, there is epidemiological evidence that the prevalence of AR is rising worldwide [3]. This may derive from the recent increase in airborne pollution, in dust mite populations, as well as other agents, or even from the “hygiene hypothesis” [1,4].

Most cases of allergic rhinitis are not extraordinary, yet the patients’ experiences provide a unique opportunity for health care practitioners and researchers to more fully understand its impact on patients’ health. Significantly, AR has been described as a disease that may appear quite bearable to the nonsufferer [5]. This alternative case report presents a fairly typical case of an adult (the second author) with ARC. However, rather than presenting laboratory, or other, findings, we use her interview to highlight her experiences and journey with allergic rhinoconjunctivitis, as well as her totally unexpected therapy and prevention using lemon.

## Case presentation

The patient is a 63-years-old, single, Greek female who weighs 60 kilograms (weighed 48 kilograms in 1981) and is 1.50 meters tall. She is consultant of preschool education and distinguished poet. She characterizes her current health condition as *“excellent”*. According to her medical case history, she had generalized ARC from 1981 (period of first diagnosis) to 1984, whereupon she was almost cured. Other health issues include primary infection by Mycobacterium Tuberculosis, without manifesting symptoms of tuberculosis, just after the appearance of ARC (1981), detection of helicobacter pylori, gastro-esophageal reflux (2003), chronic gastritis (2003), hypercholesterolemia (2003). She also underwent laparoscopic cholecystectomy (2003) after the diagnosis of acute cholocystitis. Her current medication comprises ezetimibe/simvastatin (2005) and esomeprazole magnesium (2006). She has a family history of cancer (brother, cousin, uncle) and gastro-esophageal reflux (father), but no family history of allergies. She reported neither drug nor alcohol abuse. She had been smoking 3-6 cigarettes daily from 1966 to 1996 and has been drinking 1-2 cups of coffee daily. She exercises (walking, dancing) five times a week for an hour each time.

She first developed ARC in 1981 while working in the village Katsikas near the city of Ioannina. She reports that she developed, and still has, hypersensitivity mainly to washing powder, as well as to most kinds of detergents (chlorine water, ammonia-containing solutions), every kind of dust, house dust mite and dust produced by road-traffic, gas fumes, air conditioning, pollen, scents of flowers, blossoms and plants, perfumes and synthetic aromatics. She cannot remember the exact stimuli or date of the first allergy symptoms. Describing her case, she emphasizes that her symptoms appeared all year round, not in a certain season, everywhere she was, even when she travelled somewhere or during a journey. ARC occurred after her exposure to the afore-mentioned allergens and had no connection with periods of stress or emotional discomfort. It popped up and went away, popped up, went away! “I was impressed! But, it followed me even in Russia! It was not present only in Greece. I thought that something was wrong with Greek environment!” The symptoms continued for days, weeks, or even several months and did not subside even if the allergen had retreated. She had intense watery rhinorrhea, tearing and irritation of the eyes, as well as repetitive sneezing. She also had pain, erythema and unbearable pruritus of the nose, pharynx and eyes. “Acute crises! Crises! Recurrent crises!” It was terrible! She mentioned no cough, headache, impaired smell, or nasal congestion. She said she performed allergy testing (1982) which revealed the probable allergen she was reacting to: white poplar of Belarus. “And I said that we don’t have such kind of poplar here!”

She was treated with corticosteroid pills (She cannot remember the exact name of the drug) and nasal decongestant sprays. In periods of acute exacerbation, she took the corticosteroid pills permanently. This medication pattern continued for three years. Although it was really effective in considerably reducing the allergy symptoms, the patient mentions that the action of cortisone begun only after three hours. Moreover, she noticed that she started to gain weight at the abdominal regions. This resulted in her low compliance to cortisone-including medication. “I suddenly started to gain weight. The pills contained cortisone and my doctor hadn’t informed me so.”

Though, it is highly important to evaluate the impact of her illness on her life. For her it was a painful experience, which impaired her total well-being. She had to confront recurrent crises and she often was not able to sleep at night. The symptoms were intense and she had severe physical discomfort. She was unable to work, to talk with friends, to have social life when her condition was aggravated. “I couldn’t go for a coffee and talk with friends.” Her productivity and concentration were severely restricted. “I could think of nothing else.”

The solution to her problem came really unexpectedly for her. It was a period of crisis in 1984, when she also had nausea and a feeling of stomach discomfort. Thus, she tried to drink lemon juice, hoping it would improve her abdominal pain (popular traditional therapy in Greece). Oddly enough, She noticed that her coexisting allergy symptoms improved radically! She tried it again and again and again; and in all times the result was the same. From that moment, lemon became an integral part of her life. Every time she felt that her allergy symptoms come up, she expressed juice from half or a whole lemon (half if her symptoms were relatively mild, a whole one when she was in crisis) and, then, she diluted it with a little water. Lemon brought a total change to her illness and life. Her symptoms’ intensity and incidence decreased significantly. Lemon worked in only half an hour and directly relieved her crisis. She comments that only lemon juice had this outcome. She had been drinking orange juice in rich quantities but it never alleviated the vigour of her symptoms. The consumption of lemon juice reduced her rhinorrhea, which started to give its place to a mild nasal congestion. It also reduced tearing of the eyes, sneezing, pain and pruritus. The fact is that she no longer had, nor has, crises.

## Discussion

Allergic rhinitis is a major chronic respiratory disease of high prevalence. The prevalence of self-reported AR is estimated to 18.7% across Europe [6], and 14.2% across the United States [7]. In addition, it can be a debilitating condition which, if untreated, can result in considerable health-related and economic consequences. It causes a significant impairment in quality of life of patients, particularly in general health, vitality, bodily pain, social functioning, role emotional and mental health, especially in women [8], along with high direct, indirect, hidden and out-of-pocket costs for patients and health-care system [1]. Cost and damage to the health of patient may increase in cases of poorly controlled allergic rhinitis. These may lead to or co-exist with other nasal or sinus diseases, such as asthma, conjunctivitis, rhinosinusitis, nasal polyps, adenoid hypertrophy, tubal dysfunction, otitis media with effusion, chronic cough, laryngitis, gastro-esophageal reflux [2].

According to studies, such as “Allergic rhinitis management pocket reference 2008” and “ARIA 2008” [2,9], we could classify her case as severe allergic rhinoconjunctivitis, with periods of remission of her symptoms. She showed very low compliance to cortisone therapy and this is reasonable because the more a certain type of medication inhibits the performance of daily activities, negatively affects the physical functioning and identity issues of the patient, the less is the patient willing to carry it out successfully [10]. Lemon came to her life as a miracle, to change her illness’ routine. No drug can alleviate her symptoms except for lemon, which is not a drug but a citrus fruit. Thus, there is a clear causal association between lemon juice and her illness’ therapy [11]. “It’s something utterly necessary in my life.”

Former studies [Medline was searched using the algorithm (rhinitis OR conjunctivitis OR asthma) allergic lemon on 27 March 2009] have supported citrus fruits’ potential inhibitory effect on the pathophysiological mechanisms of IgE-mediated allergic disorders. Kobayashi & Tanabe provide some evidence that Citrus unshiu powder inhibits histamine release from basophils of patients suffering from seasonal allergic rhinitis to cedar pollen: among the flavonoids in Citrus unshiu powder, hesperetin and nobiletin inhibited the degranulation of RBL-2H3 cells via, at least in part, suppression of PI-3 kinase activity [12]. Another possible biological mechanism suggests that Citrus/Cydonia compilation is likely to induce more regulatory T-cells, whether CD4+CD25+Fosp3+ natural or antigen induced IL-10 and/or TGF-b producing Tr-cells, that are, therefore, very immunosuppressive, and which are capable of reducing allergen specifically activated Th2 cells [13].

## Patient’s perspective

**Early days** The periods of exacerbation were not for a while but lasted long, even months! I had awful itching of my face, particularly of the nose. It was so acute that I almost poked my fingers at my nose. It was terrible! My eyelids were fluttering and I had always that sneezing, not once, but repeatedly, in gusts. It was awful! My nose was aching and was red, I couldn’t talk. Crises! Crises! Recurrent crises!

And it was my work that I had to be with kids. It was very difficult! When I was about to organize a seminar, I had to undergo therapy in order to prevent a possible crisis. Thus, I took pills permanently Oh! I didn’t want to take pills at all, I didn’t take even an aspirin and the fact that I had to, ruined my psychological well-being. I couldn’t bear it. I thought I was too young to take pills.

When I was sick, I couldn’t sleep. On the other hand, my people really suffered with me. They often said what is that girl going through, what are we going to do. There were nights I was in great crisis. It was very painful. I went crazy! I felt like I was dying.

It was impossible to go out. My problem was predominant in my life. I always wiped with endless napkins! You couldn’t live like that, it was isolation! I didn’t want to see anybody, nor for anybody to see me. I just wanted to disappear!”

**Later days** “When I was working in Messolongi (a town 248 kilometres from Athens) in 1984, I had a problem with my stomach; I had nausea. So, I tried lemon and this did me good. I felt that my stomach improved and, at the same time, my allergy started fading! I expressed juice from a lemon.

If my symptoms were intense, I drank juice from one lemon. If they were mild, juice from half a lemon. My crises are now reduced very much! They’re not as frequent (they may come up 2-5 times a year) and intense as they used to.

I always carried lemons with me, at all my journeys, even on vacation! I may have forgotten my toothbrush, or anything else, but no way the lemons! I always carried, and still carry, a knife and a lemon! It’s something utterly necessary in my life. It’s something I never want to miss. It will always be in my apartment. I know that I can weather any crisis with a lemon! With a lemon or a great love story!

For me, lemon blossoms are not only a wedding symbol, but also a broad symbol of Greek culture. As a little kid, in my village, I saw how people used them to make the wedding wreath and the brides wearing them. Yet, I love the leafage, this magnificent dark-green leafage, which is smooth and shiny and its perfect scent! I believe that lemon, among all citrus fruits, and the lemon blossoms of course, is something beloved to me! And to poetry as well! Lemon completely solved my problem! I have been living 25 years without this illness! I’m too happy.

## Abbreviations

AR: allergic rhinitis
ARC: allergic rhinitis and allergic conjunctivitis
